# Editorial: Intersectional perspectives on the lived experiences of people with intellectual disabilities across the lifespan

**DOI:** 10.3389/fpsyt.2026.1985540

**Published:** 2026-09-25

**Authors:** Mary Wickenden, Callista Kahonde, Fleur Boot

**Affiliations:** 1 Institute of Development Studies, Brighton, United Kingdom; 2 Stellenbosch University, Stellenbosch, South Africa; 3 Erasmus Universiteit Rotterdam, Rotterdam, Netherlands

**Keywords:** adults, global perspectives, intellectual disabilities, intersectionality, lifespan, lived experiences, qualitative

## Introduction

This Research Topic brings together research and scholarship that critically examines how the intersection of multiple identities influences the lives of people with intellectual disabilities across the lifespan. Over the years, intersectionality has increasingly been used as a critical framework for analysing the lived realities of people with disabilities. Originally coined by Crenshaw (1989), intersectionality provides a lens for understanding how the interaction of multiple identities, such as age, gender, race, culture, sexuality, socioeconomic status, disability, and geographical location, shapes individuals’ lived experiences. Although people with intellectual disabilities often experience overlapping forms of marginalization, stigma, and discrimination throughout their lives, these disadvantages have rarely been explored through an intersectional lens. Instead, other aspects of identity are frequently overshadowed by focussing on the intellectual disability itself.

The six papers in this Research Topic are drawn from diverse contexts and collectively demonstrate how the lives of people with intellectual disabilities are shaped by the intersection of disability with other usually overlooked identities. These include sexuality, living arrangements, sexual and reproductive health, housing, humanitarian settings, and the skills and perspectives of service providers. Together, the papers underscore the importance of moving beyond single-axis understandings of disability to recognise the complexity of people’s lived experiences and the multiple, intersecting factors that shape their opportunities, challenges, and wellbeing across the lifespan.

Although we have chosen to use the term intellectual disability in the title of this Research Topic, some authors use alternative terminology, including learning or cognitive impairment, difficulty or disability. These terms are used according to the authors’ respective disciplinary, cultural, or national contexts and broadly refer to the same population.

## Overview of the Research Topic

Stanojevic et al. explore the intersection of intellectual disability with sexual and gender diversity, focusing on LGBTQ+ individuals with intellectual disabilities and the professionals who support them in the US context. The authors examine how knowledge, affirming attitudes, and direct experience influence professionals’ confidence in providing LGBTQ+-affirmative services. They highlight the unique challenges faced by individuals whose experiences are shaped by both disability and LGBTQ+ identities, while demonstrating the importance of intersectionality-informed training and practice in fostering inclusive environments.

Thurman et al. turn our attention to the intersection of intellectual disability and homelessness also in the US. Through the perspectives of service providers, the paper reveals how adults with intellectual disabilities must navigate multiple and often poorly coordinated systems in order to access housing, healthcare, and social supports. The findings expose the ways in which structural barriers, policy gaps, and systemic ableism can compound disadvantage, reinforcing cycles of exclusion and instability for some of society’s most vulnerable individuals.

Andualem et al. examine the lived experiences of women with intellectual disabilities in Ethiopia as they seek access to sexual and reproductive health services. Here, disability intersects with gender, culture, poverty, and social norms to shape opportunities and constraints. The paper provides insight into barriers related to health education, contraception, protection from abuse, and reproductive decision-making, while also identifying facilitators that promote greater inclusion and autonomy. The voices of the women themselves offer a powerful reminder of the importance of culturally responsive and disability-inclusive healthcare systems.

Abrahams and Kleintjes broaden the discussion to intersectional analysis of respite care in South Africa. Focusing on people with intellectual disabilities and their caregivers, the authors demonstrate how disability is experienced within broader systems of race, gender, socioeconomic inequality, geography, and historical disadvantage. Particular attention is given to the experiences of poor Black families and female caregivers, whose caregiving responsibilities are often intensified by persistent structural inequities. The paper illustrates how multiple forms of marginalization converge to influence access to support and quality of life.

Rohwerder considers the experiences of people with intellectual disabilities during conflicts, natural hazard-related disasters, and public health emergencies with a global perspective. Considering crises through the lens of intellectual disability, she underlines how existing inequalities are often magnified in times of upheaval. Disruptions to support networks, inaccessible information, and exclusion from preparedness and recovery efforts place individuals with intellectual disabilities at heightened risk. The paper highlights the need for disability-inclusive approaches to emergency planning and humanitarian action that recognize and address these intersecting vulnerabilities.

Gur turns attention to a less visible but deeply significant dimension of lived experience: the cumulative effects of stigma, marginalization, and social devaluation. Introducing the concept of insidious trauma, the paper argues that people with intellectual disabilities may experience chronic psychological harm arising from persistent experiences of exclusion and identity-based oppression. By expanding traditional understandings of trauma beyond discrete adverse events, the paper provides a framework for understanding how social inequalities become internalized and affect well-being throughout the life course.

## Conclusion

The papers underscore the importance of intersectionality as both an analytical framework and a call to action. Whether examining sexuality, housing insecurity, gender, caregiving, crisis experiences, or trauma, each contribution demonstrates that the lives of people with intellectual disabilities are shaped by the interaction of multiple identities and social forces. Taken together the Research Topic challenges researchers, practitioners, and policymakers to move beyond narrow understandings of intellectual disability and toward approaches that acknowledge complexity, address structural inequities, and support the full participation and well-being of people with intellectual disabilities across the lifespan in different socio-economic and cultural contexts.

The response to our call for papers considering intersectional aspects of the lives of people with intellectual disabilities, has been encouraging. However, it leaves many topics uninterrogated. This perhaps reflects the historical and present dearth of research about and with this subsection of the disabled population globally, but more particularly in low- and middle-income countries. In these contexts, they are habitually stigmatized and excluded from research. There is a still a tendency towards clinical and impairment focused research and interventions, rather than thinking about people’s lives using a broader lens. Recognition of both the ordinary and extraordinary needs of people with intellectual disabilities, through increased research about them and with them is urgently needed. We hope that this Research Topic will contribute to increased awareness and further discussion.

